# Predictors of perioperative morbidity in elderly patients undergoing colorectal cancer resection

**DOI:** 10.1007/s10151-024-03040-z

**Published:** 2024-11-27

**Authors:** S. Y. Parnasa, N. Lev-Cohain, R. Bader, A. Shweiki, I. Mizrahi, M. Abu-Gazala, A. J. Pikarsky, N. Shussman

**Affiliations:** 1 Department of General Surgery, Hadassah Medical Organization and Faculty of Medicine, Hebrew University of Jerusalem, POB 12000, 91120 Jerusalem, Israel; 2 Department of Radiology, Hadassah Medical Organization and Faculty of Medicine, Hebrew University of Jerusalem, Jerusalem, Israel

**Keywords:** Colorectal cancer, Perioperative complications, Elderly, Frailty, Sarcopenia, Modified 5-item frailty index

## Abstract

**Aim:**

Colorectal cancer resection in the elderly may be associated with significant morbidity. This study aimed to assess perioperative morbidity in elderly patients undergoing colorectal cancer resection and to investigate risk factors for postoperative complications.

**Materials and Methods:**

Consecutive patients aged ≥ 75 years undergoing colorectal cancer resection with curative intent between January 2014 and December 2021 at our institution were included. We evaluated risk factors for postoperative complications, length of hospital stays (LOS), 30-day readmission, and 90-day mortality rates.

**Results:**

A total of 843 patients underwent colorectal cancer resection during the study period, of whom 202 patients were 75 years or older. Advanced age was associated with postoperative complications (Clavien–Dindo score > 3b, *p* = 0.001). Sarcopenia, preoperative plasma albumin < 3.5 g/dL, and open and urgent surgery were significantly correlated with major complications (*p* = 0.015, *p* = 0.022, *p* = 0.003, and *p* < 0.001, respectively). LOS was longer in elderly patients with a modified 5-item Frailty Index (5-mFI) ≥ 2 and low preoperative serum albumin levels, as well as following open surgery (*p* = 0.006, *p* = 0.001 and *p* < 0.001, respectively). Sarcopenia and preoperative plasma albumin < 3.5 g/dL were predictors for 90-day mortality (*p* = 0.004 and *p* > 0.001).

**Conclusion:**

Advanced age, sarcopenia, preoperative hypoalbuminemia, 5-mFI ≥ 2, and open or urgent surgery may serve as predictors for postoperative morbidity in the elderly population.

## Introduction

Colorectal cancer (CRC) ranks as the third most prevalent cancer globally, with a median age of CRC diagnosis at 67 years. According to data from the National Cancer Institute, 56% of newly diagnosed patients with CRC are aged over 65 years, and 31% are over 75 years [1]. With an increasing life expectancy, a significant increase in the number of older patients with CRC is anticipated.

Surgery remains the mainstay of curative treatment for colorectal cancer. Recent studies suggest that short-term outcomes in elderly patients undergoing surgery for CRC are affected not only by chronological age, but also by factors such as frailty, sarcopenia, malnutrition, and other comorbidities, which contribute to adverse outcomes within the elderly population [2–6]. Therefore, careful consideration of these issues is imperative before opting for surgical intervention in this patient population. Although there is no globally accepted definition of “elderly patients,” chronological age has been a widely utilized variable in assessing perioperative outcome assessments. Recognizing the increasing life expectancy, the ages of 70 and 75 years have more recently been adopted as the cutoff points for defining elderly patients [7, 8].

Given the heterogeneity within the elderly patient population, accurate preoperative assessment of risk factors is crucial for improving patient outcomes. This study aimed to assess postoperative morbidity and mortality among elderly patients (≥ 75 years) following colorectal cancer resection and to identify risk factors for adverse perioperative outcomes in this population.

## Materials and methods

### Study design

In this single-center retrospective cohort study, we used a prospectively maintained database. Approval for this study was obtained from the Institutional Review Board of the Hadassah Hebrew University Medical Center (approval number HMO-0766-20), and the study was conducted in adherence to the principles of the Declaration of Helsinki. Patient records were anonymized and deidentified prior to analysis.

### Patient inclusion and exclusion

We reviewed the charts of all consecutive adult patients who underwent colorectal resection due to cancer with curative intent between January 2014 and December 2021. Both elective and urgent procedures were included. Patients aged < 75 and those undergoing surgery for indications other than cancer were excluded from the study.

### Data collection

Demographic information, comorbidities [according to the American Society of Anesthesiology (ASA) score], preoperative data (including serum albumin and hemoglobin levels), operative data (such as operative time, intraoperative complications, and surgical approach), length of postoperative hospital stay, and postoperative complications (according to the Clavien-Dindo classification [9]) were extracted from a prospectively collected CRC database. The modified 5-item Frailty Index (5-mFI) score was calculated to assess frailty. This score consists of four preoperative comorbidities and one functional variable: congestive heart failure, chronic obstructive pulmonary disease, hypertension requiring medication, diabetes mellitus requiring oral agents or insulin, and nonindependent functional status before surgery. Each variable represents one point for a total possible score of five points. A score of two or greater indicates frailty status [10].

**Fig. 1:**
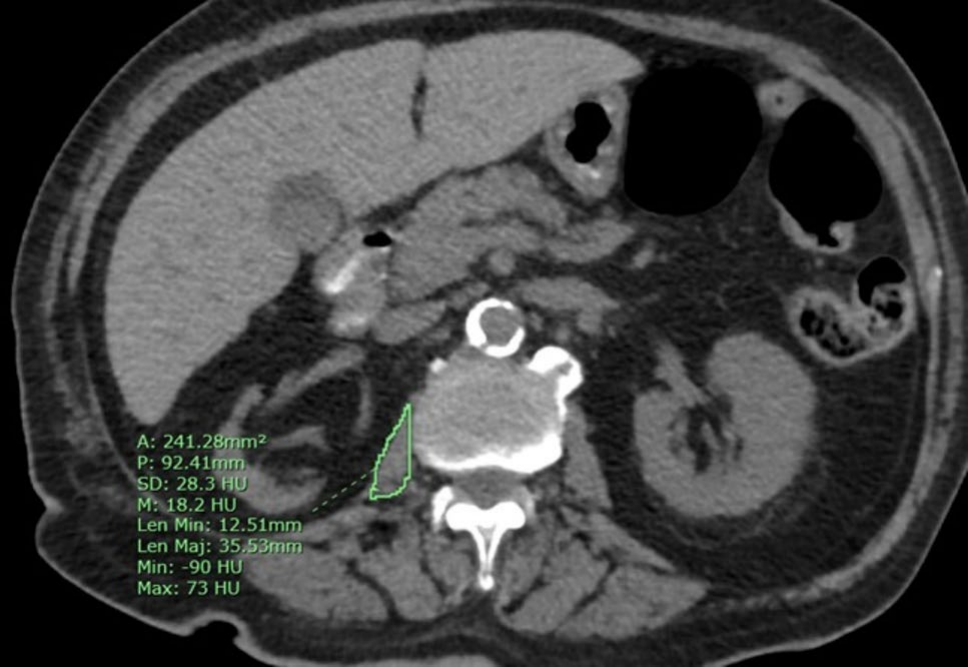
Axial image from a non-contrast preoperative computed tomography scan of an 80-year-old patient. The area of the right psoas muscle, measured at the level of L3, is 241 mm^2^ with an average density of 18.2 Hounsfield units

**Fig. 2:**
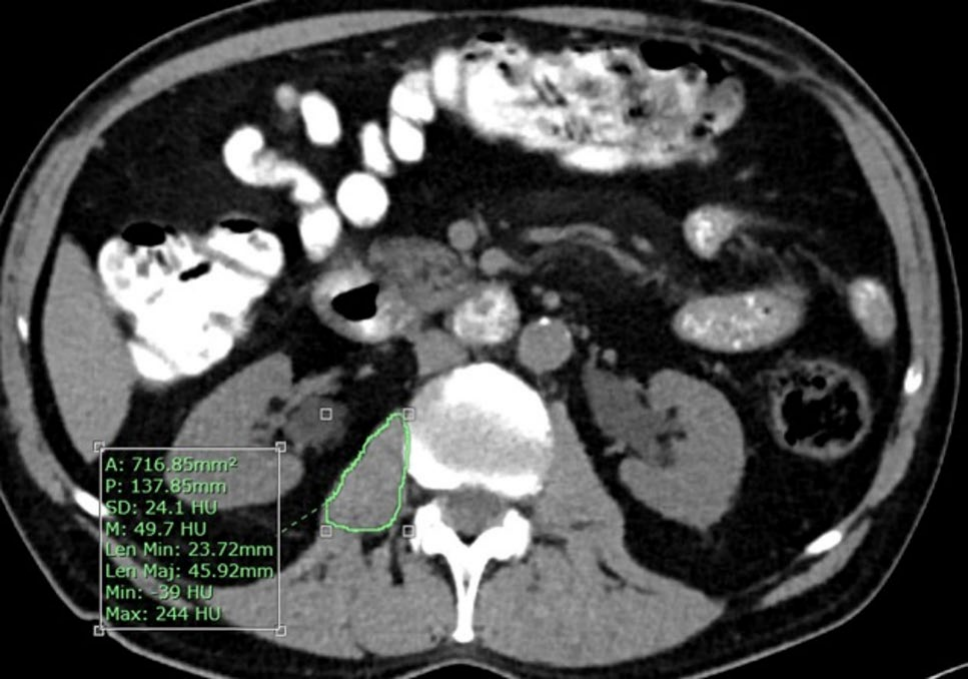
Axial image from a non-contrast preoperative computed tomography scan of a 77-year-old patient. The area of the right psoas muscle, measured at the level of L3, is 717 mm^2^ with an average density of 49.7 Hounsfield units

Sarcopenia was defined using sex-specific cutoff points for the third lumbar vertebra (L3) skeletal muscle index due to its accuracy in reflecting the real muscle mass and fat volume [11]. Muscle mass loss was quantified using several measures derived from a perioperative computed tomography (CT) scan (Figs. 1, 2):

- Psoas muscle density measured on the Hounsfield unit scale (−29 to +150) at the level of the L3 vertebra.
- Transverse psoas muscle thickness (TPMT)/height, calculated as the greatest transverse diameter of the psoas muscle (anterior–posterior oblique) proportional to the patient’s height (mm/m).
- Psoas muscle area, the sum of the product of the transverse diameter and the longitudinal diameter of the psoas muscles on both sides (cm^2^).
- Psoas muscle index (PMI), calculated as the psoas muscle area divided by the patient’s height (cm^2^/m^2^).

### Study endpoints

Outcome measures included LOS, postoperative complications, 30-day readmission, and 90-day mortality.

### Statistical analysis

A professional statistician conducted analyses using SPSS version 28.0. Continuous variables were presented as median (interquartile range, IQR) or mean ± standard deviation, while qualitative variables were expressed as frequencies and percentages.

Univariate analyses, including the independent *t*-test, Mann–Whitney *U* test, and chi-squared test, were initially employed. Subsequently, multivariate logistic regression models, using the forward stepwise selection method, were applied to test risk factors associated with postoperative complications. All statistical tests were two-tailed, and statistical significance was defined at *p* < 0.05.

## Results

### Patient and surgical characteristics

A total of 843 patients with colorectal cancer were operated on at our institution during the study period. Of these, 202 patients aged 75 years old and older.

The majority of the patients were male (55%), and 19% of the study cohort had a preoperative hemoglobin level of less than 10 g/dl, while 19% of the study cohort had preoperative hypoalbuminemia (< 35 g/L). In total, 53% of the surgeries were right hemicolectomies and 19% were urgent surgeries (Tables 1, 2).

**Table 1:** Preoperative characteristics of elderly (≥ 75 years) patients with colorectal cancer

Female, *n* (%)	91 (45.0%)
Male, *n* (%)	111 (55.0%)
ASA score, *n* (%)	
1	9 (4.5%)
2	113 (55.9%)
3	80 (39.6%)
Preoperative serum Hemoglobin	
< 10 g/dl^1^, n (%)	38 (19.1%)
≥ 10 g/dl^1^, n (%)	161 (80.9%)
Preoperative serum albumin	
< 35 g/L^2^, n (%)	32 (18.9%)
≥ 35 g/L^2^, n (%)	137 (81.1%)
Stage	
1	42 (20.7%)
2	76 (37.6%)
3	58 (28.8%)
4	26 (12.9%)

*IQR* interquartile range, *BMI* body mass index, *kg* kilogram, *m* meter, *ASA* American Society of Anesthesiologists, *n* number, *g* gram, *dl* deciliter

^1^Data were available for 199 patients

^2^Data were available for 169 patients

**Table 2:** Surgical data and postoperative outcomes of elderly (≥ 75 years) patients with colorectal cancer

Surgical procedure, *n* (%)	
Right hemicolectomy	107 (53.0%)
Left hemicolectomy	55 (27.2%)
Low anterior resection	24 (11.8%)
Abdominoperineal resection	8 (4.0%)
Subtotal colectomy	8 (4.0%)
Urgent surgery, *n* (%)	38 (19.0%)
Open surgery, *n* (%)	76 (37.8%)
Intraoperative complications, *n* (%)	
Unplanned stoma	0
Additional resection of adjacent organs	5 (2.5%)
Conversion to open surgery	11 (5.4%)
Hemorrhage	7 (3.5%)
Postoperative complications (CD classification), *n* (%)	
1	22 (10.9%)
2	43 (21.3%)
3a	1 (0.5%)
3b	6 (3.0%)
4a	5 (2.5%)
4b	1 (0.5%)
Hospital stay (days), median (IQR)	10 (7–15)
30-days readmission, *n* (%)	8 (4.0%)
Perioperative mortality (90-days), *n* (%)	2 (1.0%)

*n* number, *CD* Clavien–Dindo, *IQR* interquartile range

### Predictors for postoperative complications

Patients who had severe postoperative complications (CD > 3b) were older than those who did not have them (Table 3, *p* = 0.001). Low preoperative psoas muscle area correlated with higher rates of postoperative complications (Table 3). Patients with preoperative malnutrition, as indicated by serum albumin levels below 35g/l, exhibited a higher proportion of major complications (*p* = 0.022), but anemia was not predictive of postoperative morbidity. Comorbidities (higher ASA score) and frailty (5-mFI ≥ 2) did not predict major postoperative complications. Patients undergoing open surgery experienced a higher rate of major postoperative complications compared with those undergoing surgery via a laparoscopic approach (*p* = 0.003). Urgent surgery was also correlated with major postoperative complications (*p* < 0.001). In a multivariate analysis, advanced age and open surgery were identified as independent risk factors for postoperative major complications [odds ratio (OR) 1.28, 95% confidence interval (CI) 1.084–1.530 and OR 13.5, 95% CI 1.42–130.18, respectively].

**Table 3:** Risk factors for postoperative complications among the elderly patients

	CD ≤ 3b (*n* = 194)	CD > 3b (*n* = 8)	*p*-Value
Age (years), median (IQR)	80 (77–85)	90.5 (84.7–94)	**0.001**
5-m-FI, median (IQR)	2 (1–2)	2 (1.5–3)	**0.091**
Preoperative hemoglobin (g/dl), median (IQR)	11.6 (10.5–13)	10.8 (7.9–11.9)	**0.152**
Psoas muscle area (cm^2^), median (IQR)	10.2 (7–13.3)	6.4 (2.9–7.8)	**0.015**
Psoas muscle index (cm^2^/m^2^), median (IQR)	3.8 (2.6–5)	2.5 (1.8–5.2)	**0.321**
Psoas muscle density (HU), median (IQR)	40 (33.7–45)	36.5 (11–40)	**0.084**
ASA score, *n* (%)			**0.332**
1	8 (4.1%)	1 (12.5%)	
2	111 (57.2%)	2 (25%)	
3	75 (38.7%)	5 (62.5%)	
Serum albumin levels (g/L) < 35^1^, *n* (%)	28 (17.4%)	4(50%)	**0.022**
Open surgery, *n* (%)	69 (35.8%)	7 (87.5%)	**0.003**
Urgent surgery, *n* (%)	32 (16.5%)	6 (75%)	** < 0.001**

*IQR* interquartile range, *5-m-FI* modified 5-item Frailty Index, *g* gram, *dl* deciliter, *L* liter, *cm* centimeter, *mm* millimeter, *m* meter, *TPMT* transversal psoas muscle thickness, *HU* Hounsfield units, *n* number, *ASA* American Society of Anesthesiologists

^1^Data were available for 155 CD < 3a patients and 14 CD ≥ 3a patients

### Risk factors for longer length of stay

The median length of postoperative hospital stay was 10 (IQR 7–15) days (Table 2). Advanced age, low preoperative serum albumin levels, high 5-mFI, and open surgery were identified as predictors for longer hospital stays in univariate analysis (*p* = 0.001, *p* = 0.011, *p* = 0.006, and *p* < 0.001, respectively, Table 4). However, sarcopenia, preoperative anemia, urgent surgery, and higher ASA scores did not predict prolonged hospital stay. Multivariate analysis revealed that open surgery and 5-mFI ≥ 2 were independent risk factors for an extended hospital stay (OR 5.16, 95% CI 2.307–11.577, and OR 1.517, 95% CI 1.042–2.209, respectively).

**Table 4:** Risk factors for prolonged hospital stay among the elderly patients

	LOS ≤ 7 (***n* = 68)**	LOS > 7 (***n* = 134)**	p-Value
Age (years), median (IQR)	79.5 (76.2–83)	82 (78–85)	**0.001**
5-m-FI, median (IQR)	1 (1–2)	2 (1–2)	**0.006**
Preoperative hemoglobin (g/dl), median (IQR)	11.9 (10.2–13.2)	11.5 (10.4–12.8)	**0.351**
Psoas muscle area (cm^2^), median (IQR)	10.3 (7.8–13.4)	9.9 (6.6–13)	**0.337**
Psoas muscle index (cm^2^/m^2^), median (IQR)	3.8 (2.8–5)	3.8 (2.5–5.1)	**0.546**
Psoas muscle density (HU), median (IQR)	41 (35–47.2)	40 (33–45)	**0.229**
ASA score, *n* (%)			**0.700**
1	4 (5.9%)	5 (4.4%)	
2	36 (52.9%)	77 (57.1%)	
3	28 (41.2%)	52 (38.5%)	
Serum albumin levels (g/L) < 35^1^, *n* (%)	6 (8.8%)	22 (16.3%)	**0.011**
Open surgery, *n* (%)	11(16.2%)	66 (48.8%)	** < 0.001**

*IQR* interquartile range, *5-m-FI* modified 5-item Frailty Index, *g* gram, *dl* deciliter, *L* liter, *cm* centimeter, *mm* millimeter, *m* meter, *TPMT* transversal psoas muscle thickness, *HU* Hounsfield units, *n* number, *ASA* American Society of Anesthesiologists

^1^Data was available for 55 LOS ≤ 7 patients and 107 LOS ≥ 7 patients﻿

### Risk factors for readmission (≤ 30-day) and for mortality (≤ 90-day)

A total of eight (4.0%) patients required early readmission. Low psoas muscle density predicted a higher readmission rate within 30 days of surgery (*p* = 0.025), whereas other measures of sarcopenia, frailty, malnutrition, comorbidities, and surgical approach were not associated with readmission (Table 5).

**Table 5:** Risk factors for 30-days readmission among the elderly patients

	Readmission		p-Value
	No (n = 194)	Yes (n = 8)	
Age (years), median (IQR)	80.5 (77–85)	82.5 (78.2–89.5)	**0.334**
5-m-FI, median (IQR)	2 (1–2)	1.5 (1–2)	**0.855**
Preoperative hemoglobin (g/dl), median (IQR)	11.6 (10.5–13)	11.9 (10.0–13.2)	**0.918**
Psoas muscle area (cm^2^), median (IQR)	9.9 (6.7–13.2)	11.8 (6.2–14.5)	**0.738**
TPMT/height (mm/m), median (IQR)	8.5 (6.6–10.9)	9.6 (6.1–10.3)	**0.842**
Psoas muscle index (cm^2^/m^2^), median (IQR)	3.8 (2.6–5.0)	4.4 (2.4–5.2)	**0.656**
Psoas muscle density (HU), median (IQR)	40 (34–45.2)	33.0 (24–39)	**0.025**
ASA score, *n* (%)			**0.423**
1	8 (4.6%)	1 (12.5%)	
2	108 (55.4%)	5 (62.5%)	
3	78 (40%)	2 (25%)	
Serum albumin levels (d/L) < 35^1^, *n* (%)	29 (14.9%)	3 (37.5%)	**0.126**
Open surgery, *n* (%)	72 (36.9%)	5 (62.5%)	**0.157**

*IQR* interquartile range, *5-m-FI* modified 5-item Frailty Index, *g* gram, *dl* deciliter, *cm* centimeter, *mm* millimeter, *m* meter, *TPMT* transversal psoas muscle thickness, *HU* Hounsfield units, *n* number, *ASA* American Society of Anesthesiologists

^1^Data were available for 7 readmitted patients and 162 non-readmitted patients

During the study period, two patients died within 90 days of surgery. This mortality rate is too low to conclude regarding risk factors.

## Discussion

In this study, we evaluated risk factors for adverse outcomes in elderly patients undergoing colorectal cancer resection. We found that sarcopenia, preoperative hypoalbuminemia, urgent surgery, and surgery via an open approach were predictors for significant postoperative complications. High modified frailty index (5-mFI ≥ 2), hypoalbuminemia, and open surgery were risk factors for a longer hospital stay, and low psoas muscle density was a predictor for a higher readmission rate within 30 days of surgery.

The increase in the world population and life expectancy in recent decades has led to a dramatic aging of the population. According to the United Nations Population Fund, life expectancy has globally risen from 64.8 to 70 years over the past 20 years. Moreover, by 2050, people aged ≥ 60 years will account for almost 22% of the world’s population, reaching over 2 billion people [12]. Because the incidence of CRC is higher in patients aged ≥ 65, the number of elderly patients with CRC is expected to increase markedly. One of the most prominent clinical characteristics of elderly patients with CRC is the higher incidence of right-sided colon cancer compared with young patients. This incidence increases with patient age, reaching approximately 50% in patients with CRC aged ≥ 80 [13, 14]. Our study results are consistent with these data, revealing a higher proportion of right hemicolectomies among the elderly population.

We identified a high rate of urgent surgeries and advanced stage, possibly attributed to age-related variability in recognizing symptoms, seeking medical advice, primary-care referral patterns, and lack of screening in the elderly population [15]. According to Moreno et al., only 4% of CRC cases in elderly patients (> 75 years) were diagnosed following screening colonoscopy, compared with 14% in younger patients (50–75 years) [16]. These results may suggest that CRC screening should still be considered among elderly patients without major comorbidities.

While intraoperative complications do not appear to be more frequent among elderly patients with CRC [4, 5, 14], there is no clear consensus regarding the influence of age on the incidence of postoperative complications. Some authors have reported more systemic postoperative complications (respiratory, cardiovascular, renal, and infectious complications, among others) and a longer time to recover bowel function following surgery among elderly patients [17]. Our study supports these conclusions, as advanced age was correlated with major postoperative complications and prolonged LOS.

The impact of frailty on postoperative outcomes after colorectal surgery has been increasingly discussed over the past few years. Frailty is a state of vulnerability characterized by poor resolution of homeostasis following a stressor and/or the result of cumulative decline across multiple physiological systems as a consequence of biological aging [18, 19]. Several recent reviews have concluded that frailty is a strong predictor of poor outcomes following surgery [20–22]. The multicenter GOSAFE study prospectively collected geriatric patients (aged ≥ 70) undergoing major surgery for solid cancer from 26 centers worldwide. It aimed to obtain prospective data on both quality of life and functional recovery after surgery. This study highlighted the high frequency of frailty, disability, and lack of independence in activities of daily living among the geriatric patient population, emphasizing the crucial role of frailty assessment not only in predicting postoperative complications, but also in correlating with quality of life and functional recovery after surgery. [23–25].

Various tools may be used to evaluate frailty, including the existence of comorbidities, cognitive status, nutritional status, functionality, and physical performance. Consequently, the prevalence of frailty in older individuals with CRC undergoing surgery ranges between 25% and 46% [26]. One of the most widely used frailty assessment tools is the frailty index from the Canadian Study on Health and Aging (CSHA) [27]. In 2013, Tsiouris described an edition of the CSHA frailty index—the modified Frailty Index (11-mFI)—which was created by mapping the variables in the American College of Surgeons National Surgical Quality Improvement Project (NSQIP) database used to calculate the CSHA score [28]. Recently, a 5-item modified frailty index (5-mFI) has been developed and validated [10, 29, 30]. The 5-mFI (which consists of four preoperative comorbidities and one functional variable as mentioned above) may be a useful tool for evaluating the potential impact of frailty on outcomes following surgery, providing a quick and reliable preoperative assessment. Higher frailty index scores were previously shown to be associated with a greater risk of adverse postoperative outcomes, such as severe complications, longer hospital stays, higher readmission rates, and decreased long-term survival [20, 31]. Although the pathophysiological changes underlying and preceding frailty are not clearly understood, an exaggerated systemic inflammatory response seems to play an important role in this condition [32]. In this study, we found a significant correlation between frailty and longer hospital stay, but not with adverse postoperative complications.

Another hallmark of frailty is sarcopenia, defined as an age-related, involuntary loss of skeletal muscle mass and strength. Sarcopenia, which can be calculated on the basis of abdominal CT scans routinely performed for staging work-up before surgery for CRC, has been shown to have a negative impact on surgical outcomes and survival in patients with CRC [33–35]. Measurement of the psoas muscle at the L3 vertebra level enables the evaluation of the patient’s body composition and is considered a reliable way for the identification of sarcopenia before surgery [11, 34, 35] (Figs. 1, 2). Margadant et al. reported that in a study of 373 patients, major complications after CRC surgery were more frequent in the sarcopenic group. In that study, sarcopenia diagnoses were based on psoas muscle thickness measurement at the L3 vertebral level [36]; we adopted this method in the present study. Other studies also demonstrated the correlation between preoperative sarcopenia and an increased rate of postoperative complications, longer LOS, and poor prognosis among patients with CRC [37–39]. However, Pędziwiatr et al. showed that functional recovery after laparoscopic colorectal cancer surgery was similar, regardless of the presence or absence of sarcopenia [40]. In our study, sarcopenia was associated with an increase in the rate of major complications (Clavien–Dindo grade > 3b) and readmissions but did not lead to a significant increase in LOS. Sarcopenia may lead to altered systemic inflammatory response and endocrine function and can reflect poor nutritional status and insulin resistance [41, 42]. These mechanisms, along with postoperative functional impairment and disability, may contribute to an increase in postoperative morbidity [43]. Sarcopenia might be (but is not necessarily) the outcome of the increased metabolic activity of aggressive tumor biology, leading to systemic inflammation and muscle wasting [44]. In such cases, this could be the explanation for why sarcopenia is a poor prognostic factor in patients with CRC.

The prevalence of malnutrition in the cancer patient population ranges from 20% to 70%, with differences attributed to patients’ age, cancer type, and stage [45, 46]. Among the elderly population, in addition to frailty and sarcopenia, malnutrition has also previously been described as a risk factor for mortality, functional decline, and poor treatment response [46]. Malnutrition might result from a combination of malignant disease progression, host tumor responses, chemotherapy-related side effects, and the direct effects of intestinal obstruction and malabsorption [47–50]. Malnutrition might impair various functions, such as immunity, digestive tract function, and wound healing [51]. Deficiencies of these functions increase the risk of infection and postoperative complications [52, 53]. Moreover, immune suppression leads to inadequate antitumor immunological response [54]. Hypoalbuminemia is a known indicator of both poor nutritional status and inflammation [55], while systemic inflammation is associated with poor prognosis of cancer [56]. In this study, we found that preoperative hypoalbuminemia was a predictor for postoperative complications, and as a result, for a longer LOS.

A minimally invasive approach to CRC surgery is beneficial for elderly patients, even those with comorbidities and decreased physical activity. Short-term advantages include less intraoperative blood loss, faster recovery of bowel function, and shorter LOS, as in younger patients [57–59]. Kennedy et al. also reported that open surgery was one of the factors associated with an increased risk of complications in a multivariate analysis using the database of the American College of Surgeons for elderly patients with colon cancer [60]. In our study, open surgery was significantly associated with major postoperative complications and a longer LOS.

### Study limitations

There are several limitations to this study. First, it was a single-center, retrospective investigation. Secondly, this study did not incorporate an assessment of postoperative quality of life and survival.

## Conclusions

Aging stands out as a crucial factor necessitating consideration in determining a comprehensive treatment strategy for CRC. Preoperative assessment using a modified frailty index, along with assessments for malnutrition and sarcopenia in elderly patients undergoing CRC surgery, may help reliably predict postoperative complications.

## Data Availability

The datasets generated and analyzed during the current study are not publicly available due to participants’ privacy.
